# A Nation-Wide Evaluation of Suboptimal Lipid-Lowering Treatment Patterns Among Patients Undergoing Intervention for Acute Coronary Syndrome in Hungary

**DOI:** 10.3390/jcm13216562

**Published:** 2024-10-31

**Authors:** Gergely Gyorgy Nagy, Laszlo Mark, Andrea Gerencser, Istvan Reiber, Norbert Kiss, Gyorgy Rokszin, Ibolya Fabian, Zoltan Csanadi, Istvan Karadi, Daniel Aradi, Laszlo Bajnok, Gyorgy Paragh

**Affiliations:** 1 Centre for Cardiovascular Diseases and Internal Medicine, Borsod-Abauj-Zemplen County Central Hospital, University Teaching Hospital, Szentpeteri kapu 72-76, 3526 Miskolc, Hungary; 2 Doctoral School of Health Sciences, University of Debrecen, Nagyerdei krt. 98, 4032 Debrecen, Hungary; 3 Department of Cardiology, Békés County Central Hospital Pándy Kalman Branch, Semmelweis u. 1, 5700 Gyula, Hungary; dr.mark.laszlo@gmail.com; 4 Novartis Hungary Ltd., Bartók Béla út 43.47, 1114 Budapest, Hungary; drgerencserandrea@gmail.com (A.G.); norbert.kiss@novartis.com (N.K.); 5 Department of Medicine, St George University Teaching Hospital of Fejér County, Seregélyesi út 3, 8000 Székesfehérvár, Hungary; reiberistvan60@gmail.com; 6 RxTarget Ltd., Bacsó Nádor u. 10. fsz. 2, 5000 Szolnok, Hungary; rokszin.gyorgy@rxtarget.hu (G.R.); fabian.ibolya@rxtarget.hu (I.F.); 7 Department of Cardiology, Faculty of Medicine Debrecen, University of Debrecen, Móricz Zsigmond krt. 22, 4032 Debrecen, Hungary; drcsanadi@hotmail.com; 8 Department of Internal Medicine and Haematology, Semmelweis University, Üllői út 26, 1085 Budapest, Hungary; karadi.istvan@med.semmelweis-univ.hu; 9 Balatonfüred State Heart Hospital, Gyógy tér 2, 8230 Balatonfüred, Hungary; daniel_aradi@yahoo.com; 10 Heart and Vascular Centre, Semmelweis University, Városmajor u. 68, 1122 Budapest, Hungary; 11 1st Department of Internal Medicine, Medical School, University of Pécs, Ifjúság u. 13, 7624 Pécs, Hungary; bajnok.laszlo@pte.hu; 12 Division of Metabolic Diseases, Department of Internal Medicine, Faculty of Medicine, University of Debrecen, Nagyerdei krt. 98, 4032 Debrecen, Hungary; paragh.gyorgy@med.unideb.hu

**Keywords:** acute coronary syndrome, lipid-lowering therapy, adherence, persistence, statins, ezetimibe

## Abstract

**Background/Objectives**: A significant gap exists between guideline recommendations and everyday practice. Stringent treatment is needed for vulnerable patients with acute coronary syndrome (ACS). **Methods**: Data on the lipid-lowering therapy (LLT), including the adherence, persistence, and mortality of patients undergoing percutaneous coronary intervention or bypass surgery in Hungary in 2018 were followed up and analyzed based on the National Health Insurance Fund database until the end of 2020. **Results**: A total of 12,997 patients underwent revascularization for ACS in 2018, whose discharge therapy included any LLT, a high- or moderate-intensity statin, or ezetimibe at a proportion of 91%, 75%, 12%, and 4%, respectively. By the end of the observation period, the frequency of ezetimibe administration increased to 11%. Persistence decreased, reaching 50% for all therapeutic regimens by month 16. Patients on moderate statin doses had a significantly higher mortality rate at the end of follow-up than those receiving high-intensity statin with (20% vs. 9%, *p* < 0.0001) or without (20% vs. 14%, *p* = 0.00029) ezetimibe. Those taking less potent statin doses had higher rates of comorbidities; for example, a minimum of three comorbidities were present in 39% of patients taking medium statin doses and 23% among those on high-intensity statin therapy (*p* < 0.0001). **Conclusions**: LLT persistence decreased during follow-up. The administration of a higher-intensity lipid-lowering regimen was associated with better persistence and adherence, along with more favorable mortality rates. Multimorbidity was associated with the use of lower statin doses. The results suggest that more attention is needed in terms of lipid control of females, elderly people, and individuals with several comorbidities, and emphasis should be placed on improving persistence and increasing the frequency of combined LLT prescriptions.

## 1. Introduction

In recent decades, lipid lowering has become a basic element of cardiovascular prevention, with numerous clinical trials demonstrating that lowering low-density lipoprotein cholesterol (LDL-C) can reduce cardiovascular events and mortality [1]. Target lipid values depend on the risk category, and basically, the higher the risk, the greater the possible rewards [2,3] and the lower the target value. In line with the results of studies published in recent years, previous LDL-C target values have been lowered by the 2019 European Society of Cardiology/European Atherosclerosis Society (ESC/EAS) lipid guidelines. Accordingly, in one of the most vulnerable and most populous patient groups of cardiology practice (i.e., those with a history of ACS), the targets of the LDL-C level to be achieved are 1.4 mmol/L (55 mg/dL), with at least a 50% reduction in LDL-C. Furthermore, a target value of 1.0 mmol/L (40 mg/dL) might also be considered in the case of a vascular event recurring within two years [1]. On top of the stricter LDL-C goals, a European clinical consensus statement emerged, which proposed the lipid-lowering strategy of “strike early and strike strong”. This suggests the immediate administration a dual lipid-lowering treatment for selected patients after ACS [4]. In the meantime, a similar expert consensus decision issued by the American College of Cardiology (ACC), focusing on lipid-lowering therapies other than statins, recommends PCSK-9 inhibitors as the preferred initial nonstatin therapy for adults with atherosclerotic cardiovascular disease (ASCVD) at very high risk on statin treatment for secondary prevention and failed to achieve a ≥50% LDL-C reduction and an LDL-C <1.4 mmol/L (55 mg/dL) [5]. Moreover, highly effective new agents, such as bempedoic acid (BA), are becoming available [6]. An appealing treatment approach would be the administration of various upfront combination therapies when the size of required LDL-C reduction to achieve target is >50% [6]. However, these new approaches are often hampered in everyday practice by the lack of availability, poor patient adherence, or cost and reimbursement issues. Real-world data (RWD) is, therefore, important to gain insights into healthcare delivery to diverse patient populations. This allows policymakers to identify gaps in current medical practice or suboptimal treatment patterns.

Despite the possible gains offered by lipid lowering, it is well known that treatment falls short of the expected levels all over the world. There are fewer patients on adequate therapy than required by guidelines. The rate of achieving target values still has considerable potential for enhancement [7].

According to a survey conducted in the USA with 600,000 patients with known atherosclerotic heart disease, almost half of the patients does not even receive LLT, and only 22.5% receive a high-intensity statin dose that would be required for their respective risk category [8,9]. When the European DaVinci (EU-Wide Cross-Sectional Observational Study of Lipid-Modifying Therapy Use in Secondary and Primary Care) study assessed the quality of LLT in 18 countries, the LDL-C target value of 1.4 mmol/L (55 mg/dL) was achieved in less than one-fifth of patients [9]. The situation is even less favorable in Central and Eastern European countries: target values are achieved at least 10% less frequently when compared to Western and Northern European data [10].

In a single-center Hungarian study of post-ACS patients, the rate of achieving the LDL-C target value of 1.4 mmol/L (55 mg/dL) was found to be about 20% [11], while in the study Treatment To Target with Rosuvastatin or Free/Fix Combination of Rosuvastatin and Ezetimibe for Vascular Protection in Hungarian Hypercholesterolemic PatienTs (3T-FIGHT) involving 3000 very high-risk patients, LDL-C levels below 1.4 mmol/L (55 mg/dL) could be achieved only in 11% with statin monotherapy and in 22% with a combination of a statin and ezetimibe [12].

The present study (HURDLE—HUngarian Retrospective analysis of epidemiology of atherosclerotic cardiovascular Diseases, and Lipid lowering clinical practicE) was carried out with patients who had ACS in 2018. It involved an analysis of LLT and mortality from the index event until the end of 2020, using the National Health Insurance Fund database (NHIFD). The NHIFD covers the entire population of Hungary, ensuring a complete dataset for researchers. This comprehensive coverage allows for population-wide studies and the assessment of health trends across different demographics. We investigated what type of LLT was administered to the patients after post-ACS revascularization and whether it had any impact on adherence or mortality.

## 2. Materials and Methods

### 2.1. Patient Population

This study enrolled patients based on International Classification of Diseases (ICD) codes who underwent primary PCI or coronary artery bypass grafting (CABG) surgery for ACS in 2018, had not been treated previously for this diagnosis, and were still alive beyond 30 days after the diagnosis of ACS. All consecutive patients were included in the analysis, who were registered in the NHIFD in 2018. This covered the entire population of Hungary. The acute coronary event included ST-elevation, non-ST-elevation acute myocardial infarction, and unstable angina (STEMI, NSTEMI, and UA). Death within 30 days or medically managed ACS patients were excluded from the analysis as described above. No other exclusion criteria were applied. In total, 12,997 such patients were identified. Follow-up lasted until 31 December 2020.

The NHIF identification number of this study was as follows: I043/19-4/2020 and I043/35-3/2021. The protocol was reviewed and confirmed by the Ethics Committee for Clinical Pharmacology of the Hungarian Medical Research Council (protocol code, reference number and date of approval: PMA550, OGYÉI/56698-2/2021, 13 October 2021).

### 2.2. Assessment of Endpoints

The primary endpoint of this study was all-cause mortality of the enrolled post-ACS patients. Mortality data were obtained from the NHIFD via the State Population Registration Office (SPRO) operated by the Ministry of Interior. The task of SPRO was defined by law and included documenting the place and exact time of death of individuals living in the territory of the Republic of Hungary. Important secondary endpoints were patient adherence and persistence to the prescribed lipid-lowering therapies, as well as the course of LLT according to the purchase of prescriptions (therapy initiation and dose escalation). Endpoints were stratified according to the intensity of LLT. Statin intensity was defined based on the American College of Cardiology and American Heart Association (ACC/AHA) classification of statin dosing and intensity [13]. Briefly, the high-intensity statin group included rosuvastatin 20–40 mg and atorvastatin 40–80 mg, while the moderate-intensity group included atorvastatin 10–20 mg, rosuvastatin below 20 mg, simvastatin 20–40 mg, and fluvastatin 80 mg. The more detailed definition of LLT intensity is presented in Table 1. As confirmed by the purchase of prescriptions, LLT lasted until a new lipid-lowering group, or a new dose emerged. In the absence of any amendment to a specific LLT, its end date was considered as the last day when the patient still should have had medication according to our calculations, assuming the administration of 1 tablet daily.

For the adherence calculation, the number of days covered by any lipid-lowering drug within 365 days of the start of initial therapy was calculated and divided by 365. If a patient died before the end of the period, the number of days covered by the lipid-lowering drug up to death was divided by the number of days from the initial therapy to death. Persistence was defined as a 60-day grace period, which was found to be among the best-performing algorithms for outpatient medication use [14].

The following diseases documented within 5 years preceding the index event were captured as comorbidities using ICD: heart failure, peripheral arterial disease, cerebrovascular disease, diabetes, chronic pulmonary disease, hepatic disease, renal disease, gastrointestinal ulceration, tumor, tumor metastasis, leukemia/lymphoma, chronic infectious disease, connective tissue disease, alcohol use disorder, and dementia/mental condition.

### 2.3. Statistical Analysis

A two-sample t-test was used during the statistical analysis to compare continuous variables (age and follow-up) by groups, while a chi-squared test was used for the other variables when analyzing the baseline patient characteristics. The obtained *p*-values were corrected by the Bonferroni method due to multiple comparisons. Comorbidities associated with each group of lipid-lowering agents were compared using a chi-squared test, and the obtained *p*-values were adjusted. Therapeutic persistence and survival during the observation period were plotted on KM curves and analyzed using paired log rank test and adjusted *p*-values were reported according to the Benjamani–Hochberg method. For sensitivity analysis survival and persistence of patients on various intensity LLT were plotted based on gender (male or female) and age (0–59 or ≥60 years of age) as well. The results were considered significant at *p* < 0.05. Calculations were made using the statistical software R version 4.2.0 (22 April 2022).

## 3. Results

**Figure 1:**
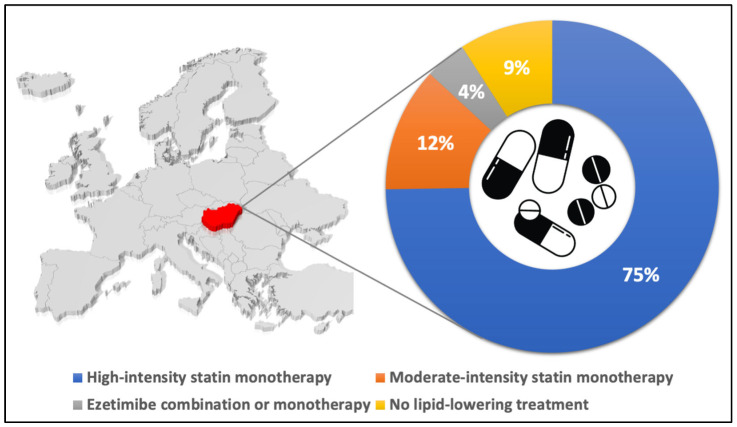
Distribution of lipid-lowering therapy after the index event in Hungarian patients with ACS in 2018.

LLT was received by 91% of the 12,997 patients who underwent PCI or CABG surgery for ACS in Hungary in 2018. Within the time window of 120 days preceding the ACS event, 27% (3218 patients) received LLT. At discharge from the hospital, most patients (75%) were on high-intensity statin, and 4% received a therapeutic regimen containing ezetimibe as well (including 1% as monotherapy and 3% in combination with high- and moderate-intensity statin) (Figure 1). During the total observational period, in the high-dose statin group, 878 patients (7%) received ezetimibe along with the statin, while in 49 patients (0.4%), combination therapy was initiated along with the reduction in the statin dose to a moderate level, and 65 patients (1%) switched from the moderate-dose statin group to the high-dose statin + ezetimibe group. By the end of the observation period, the frequency of ezetimibe administration increased to 11% from the baseline rate of 4%.

**Figure 2:**
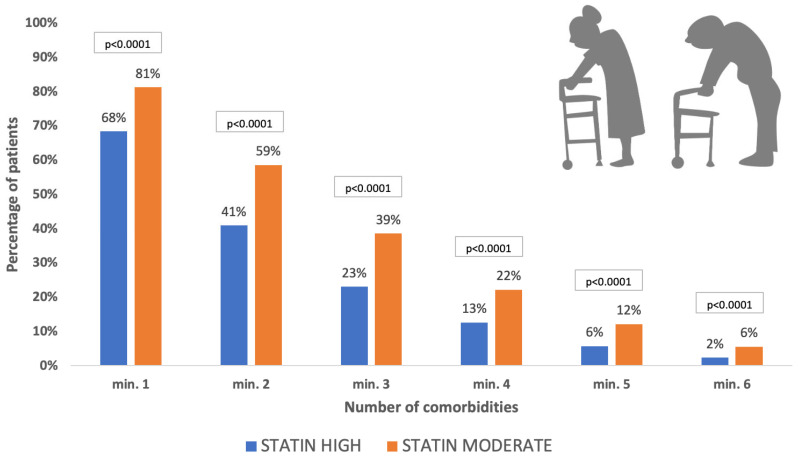
Cumulative distribution of comorbidities in post-ACS patients with high- and moderate-intensity statin dose groups.

The basic parameters, mean follow-up times, and comorbidities of patients belonging to different lipid-lowering therapy regimens are presented in Table 2. Statistical comparisons of baseline characteristics of the most used therapeutic regimens are shown on Table 3 to allow for the better interpretation of confounding parameters, such as age, gender, or comorbidities. Patients on moderate-dose statin treatment were significantly older (68.6 ± 11.68 years) than individuals in the high-intensity statin groups (64.5 ± 11.88 years, *p* = 0.001). Male-to-female ratios were lower in the groups receiving less potent LLT regimens, although this difference was only significant when comparing the moderate-dose (58.25% male) and the high-dose statin (64.35% male, *p* < 0.0001) groups. Diseases related to the cardiovascular continuum (diabetes, peripheral arterial disease, cerebrovascular disease, chronic kidney disease, and heart failure) were significantly more prevalent in the moderate-intensity statin population than in the high-intensity group (*p* < 0.0001). Similar results were found for chronic pulmonary, hepatic, and malignant diseases, these being more common among patients treated with moderate-intensity statin doses. Figure 2 presents the correlation between the number of comorbidities and a high or a moderate statin dose. In the case of one comorbidity, more patients received high-intensity statin; with the number of comorbidities increasing, a significantly higher proportion of patients received moderate-intensity statin treatment.

**Figure 3:**
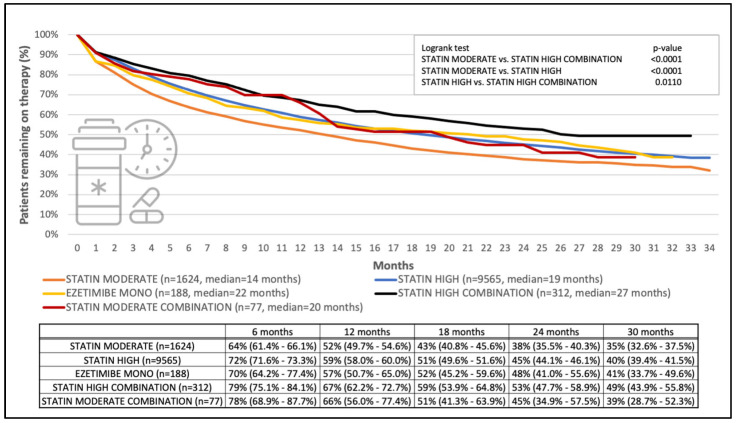
Persistence of lipid-lowering therapy in post-ACS patients across different therapeutic regimens by 60-day grace period. Selected time points are presented in numerical format (with 95% confidence intervals) for illustration.

Table 4 presents one-year adherence to different lipid-lowering therapies, as well as their associated one- and two-year persistence assuming a 60-day grace period. Figure 3 visualizes the timely course of the persistence of specific therapeutic regimens. Considering all patients and therapeutic modalities, 50% of patients took their lipid-lowering agents for 16 months; in other words, half of the patients did not take such medication beyond one year and four months. As for the overall observational period, a statistically significant difference was seen between the persistence of moderate-dose statin and high-dose statin groups (*p* < 0.0001), moderate-dose statin and high-dose combination groups (*p* < 0.0001), as well as high-dose statin and high-dose combination groups (*p* = 0.0110). The database did not allow the separation of multiple and fixed dose combinations. Comparisons by gender (male and female) and age groups (0–59 or ≥60 years of age) in the sensitivity analysis yielded consistent results (Figures S1–S4).

**Figure 4:**
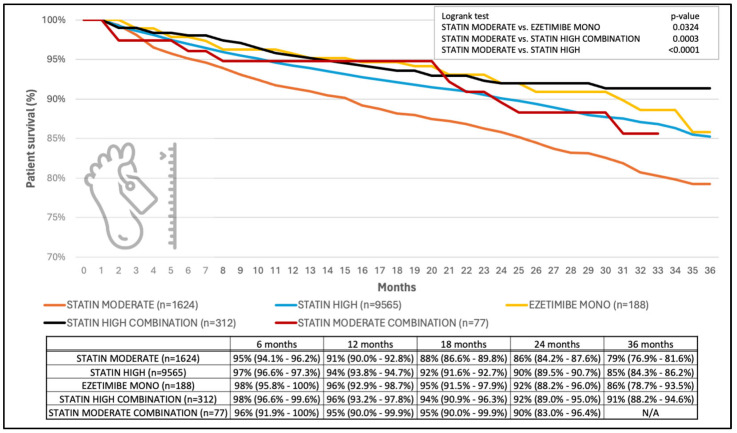
Survival analysis of patients on different lipid-lowering therapeutic regimens during the follow-up period. Selected time points are presented in numerical format (with 95% confidence intervals) for illustration.

Figure 4 presents the Kaplan–Meier survival analysis of different lipid-lowering therapeutic regimens during the observational period (lasting from the event in 2018 until the end of 2020). By the end of the study, the number of deaths and of those still alive, respectively, was 297 and 1327 in the moderate-dose statin group, 1193 and 8372 in the high-dose statin group, 26 and 286 in the high-dose combination group, 20 and 168 in the ezetimibe plus low-intensity statin group, as well as 10 and 67 in the moderate-dose combination group. Upon comparison between each group, a significant difference was observed between moderate-dose statin and high-dose statin (20% vs. 14%, *p* < 0.0001), moderate-dose statin and high-dose combination (20% vs. 9%, *p* = 0.0003), as well as moderate-dose statin and ezetimibe mono/ezetimibe + statin low (20% vs. 14%, *p* = 0.0324) groups. Mortality was lowest with a combination of high-dose statin and ezetimibe, amounting to 10% below in those taking moderate-intensity statin. The results of the sensitivity analysis for overall survival are presented in Figures S5–S8.

Survival analysis and persistence curves of patients on different lipid-lowering therapeutic regimens where a significant difference was found between the prespecified treatment groups are illustrated on Figure S9 in more detail.

## 4. Discussion

It is a well-known principle that the higher the cardiovascular risk is, the greater the possible morbidity and mortality rewards are with lipid lowering. Very high-risk post-ACS patients, therefore, require specific attention. Real-world data in this population serve several key advantages. It offers insights into how LLTs perform outside the controlled environment of clinical trials. Moreover, it helps in identifying suboptimal treatment patterns or in analyzing how healthcare delivery can affect outcomes. Concepts derived from RWD can influence policymakers and healthcare providers to optimize delivery, improve patient outcomes, focus on at-risk populations, and allocate resources more effectively. Our RWD collected during the HURDLE study was obtained from the Hungarian National Health Insurance Fund Database covering the entire population of a high-income Central European country. The research found that more intensive lipid-lowering treatment seems to be associated with better survival among post-ACS patients. The survival benefits were clearly significant for patients receiving high-intensity statin with or without ezetimibe compared to patients on a moderate-intensity statin regimen. For example, mortality was 11% lower in our patients receiving a combination therapy consisting of a high-dose statin + ezetimibe versus those only taking a moderate-intensity statin. This statistically significant difference between high- and moderate-intensity statin therapy was observed regardless of the patients’ age (Figures S6 and S7). When analyzing the prescription patterns several alerting statements could be made. First, individual comorbidities and multimorbidity were more prevalent in the moderate-intensity statin population than in the high-intensity. Second, it was more likely that older post-ACS patients were prescribed moderate-intensity statin treatment than high-intensity ones with or without ezetimibe, even though it was shown that elderly patients could clearly benefit from appropriate LLT. A systematic review and meta-analysis examining the efficacy and safety of LLT in older patients found an unquestionable reduction in the risk of major vascular events with statin and nonstatin LLT. Treatment was at least as beneficial as that seen in younger patients [15]. Third, females were over-represented among patients treated with moderate intensity statins. The male-to-female ratios were similar between the other therapeutic regimens. Several other investigators reported similar gender disparities. Females had a significantly lower probability of reaching LDL-C-recommended targets [16]. Women were less likely to receive a prescription for a statin and a higher potency LLT and/or ezetimibe [17,18], and they were more likely to discontinue LLT [18]. Our sensitivity analysis revealed that the survival difference between females on moderate- or high-intensity statin treatment regimens were not statistically significant (Figure S4). This intriguing observation cannot be explained by differences in adherence and persistence among female patients on different LLT regimens. Further studies specifically designed to address gender differences are needed.

In summary, it is very important to identify post-ACS populations who are not treated optimally to battle these unfavorable trends. For example, it has been shown by other investigators that patients who suffered an acute MI with non-obstructed coronary arteries (MINOCAs) often did not receive adequate secondary prevention therapy [19]. Altogether, these at-risk populations, therefore, require greater attention in the future to improve outcomes, such as participation in profile-specific rehabilitation programs, more frequent follow-up or personalized counseling. It had been shown that more frequent follow-up visits among ACS patients were associated with greater reductions in LDL-C levels [20]. Indeed, this is a very promising area for improving LLT. Proper and timely dose escalation is often hampered by long waiting lists and the lack of resources to increase the number of outpatient visits. This is clearly the case in Central Europe, where the public health burden of ASCVD is significantly higher than in the western or northern parts of the continent. Digital health interventions using telemedicine is an emerging new concept for offering complex cardiovascular rehabilitation for patients after an MI. Replacing personal visits with focused telehealth interventions can optimize resources and increase patient acceptance. A systematic literature review found that modern digital technology can lead to decreased LDL-C and total cholesterol in the intervention groups [21].

Other important findings of our HURDLE study were related to LLT adherence and persistence in a real-world setting. The LLT adherence and persistence of our post-ACS patients were extremely poor despite this being a highly vulnerable patient population with a strong indication for appropriate guideline-directed LLT. Every second patient discontinued lipid-lowering therapy 16 months after PCI or CABG performed due to an acute coronary event. These results were worse than reported from the literature. For example, a large systematic review and meta-analysis examined data from 3 million older statin users in 82 studies conducted over 40 countries [22]. Persistence in the secondary prevention group was 82.6% and 68.1% at 12 and 24 months, respectively, while this was between 52 and 66% and between 38 and 52% among our older patients at similar timepoints. The more intensive and combination lipid-lowering therapies were linked to better adherence and persistence in our study. For example, persistence was 38% and 54% for moderate-intensity and high-intensity statin + ezetimibe users, respectively, at two years after the ACS event. Similar results were reported by other investigators. For example, an analysis of data of almost 30 thousand patients confirmed lower cardiovascular risk with good adherence among patients on high-intensity lipid lowering [23]. It is worth noting that patients treated with high-intensity statin and ezetimibe combination therapy had the best survival and treatment persistence, even though certain high-risk comorbidities were significantly more prevalent in this group than in the high-intensity statin monotherapy population. The results on adherence and persistence were consistent between age groups and sexes as revealed by our sensitivity analysis (Figures S1–S4). The worse compliance associated with less effective LLT requires intervention, for example, the preferential surveillance of prescriptions from the National eHealth Infrastructure in this patient population.

One quality indicator of all lipid-lowering treatments is the rate of achieving the target value. This was reported to be at about 20% for 1.4 mmol/L (55 mg/dL) in previous European and Hungarian studies [9,10,11,12,24]. In the cohort analyzed in the Polish edition of the Prospective Urban and Rural Epidemiological Study (PURE), 91.8% of patients in a high cardiovascular risk group did not meet their target LDL-C criterion. In addition, 77.8% of these high-risk participants were not receiving lipid-lowering therapy at all [25]. According to the SANTORINI study, which was another European trial carried out with 9000 patients over 14 countries after the release of the 2019 guidelines, 21% of the 6401 very high-risk patients did not receive a lipid-lowering treatment at baseline. Achieving the LDL-C target of 1.4 mmol/L (55 mg/dL) occurred at a rate below 20% [24]. In the EuroPath IV study assessing LLT in patients with a history of ACS only, the rate of achieving the 1.4 mmol/L (55 mg/dL) target value was found to be 18% in 2022, compared to 10% reported in the 2018 survey. This improvement was mostly attributed to an increase in the prescription of ezetimibe from 13% to 34% [26].

However, significantly better rates could be achieved by taking appropriate care, as evidenced by the annually published results of the SWEDEHEART Swedish infarction registry. According to the 2022 summary, at the second follow-up of post-ACS patients, the target value of 1.4 mmol/L (55 mg/dL) was achieved in 41%, 52%, and 56% in 2020, 2021, and 2022, respectively. In addition to the administration of high-dose statins, a paramount factor could be that ezetimibe administration has become increasingly frequent over the years, achieving a rate of over 50% by 2022 [27]. Apparently, a key to achieving better target values is the addition of ezetimibe to a high-dose statin during more frequent, scheduled follow-up visits.

The 2021 position paper on the optimal lipid-lowering treatment for ACS, compiled by Eastern European experts mostly, claims that in this region, achieving the target lipid values is significantly hindered by the reimbursement regulations for ezetimibe and PCSK-9 inhibitors [28]. According to our HURDLE study, ezetimibe is used infrequently in Hungary. Although the reimbursement system does not facilitate its prescription (ezetimibe may only be prescribed with reimbursement 3 months after a vascular event, if an LDL-C level of 1.8 mmol/L is not achieved with a high-dose statin), even the available resources are not utilized to their fullest. While our study demonstrated an increase in its use from 4% to 11%, this still falls short from the values reported in the Swedish infarction registry or in EuroPath. Naturally, the administration of ezetimibe does not solve every lipid-lowering insufficiency, but it might yield significant improvement in target value achievement. The ILEP (International Lipid Expert Panel) Position Paper introduced the idea of the upfront lipid lowering combination therapy for the first time [24,28], and this was supported by papers of European experts [29,30,31].

In addition, providing appropriate initial LLT, emphasis should be laid on improving adherence and persistence. According to our results, half of the patients failed to take their LLT after 16 months. Discontinuation was significant in all therapeutic modalities, but either persistence or adherence was best with a combination consisting of a high-dose statin and ezetimibe. Education and a higher level of health awareness are basic options to improve our patients’ level of cooperation [32]. When a statin is co-administered with ezetimibe, using a fixed-dose combination is more suitable as it improves adherence as well [30,33]. In our study, we were not able to analyze separately the multiple and fixed dose combinations, but using the latter not only the adherence was better but also the clinical outcomes. This was shown by Polish and Korean retrospective studies on post-ACS patients [34,35]. Altogether, it is quite clear from our results that complex interventions are urgently needed on behalf of healthcare providers and policymakers to increase patient compliance for accepting lipid-lowering medical treatments. This should include regulatory measures, financial incentives, educational campaigns, behavioral and psychological approaches, access and convenience improvements, and possibly legislative actions. The measures applied should be tailored to the specificities of each society and regulatory environment where it is implemented. For example, an indicator-based performance assessment of general medical services (primary healthcare providers) has been recently introduced in Hungary. This incentivizes general practitioners to encourage patient compliance by linking their reimbursements to patient outcomes or adherence to treatment protocols. This system should include post-ACS lipid management and could be expanded towards tertiary care providers involved in the care of ASCVD patients. A recent systematic review and meta-analysis claimed that no single strategy or group of strategies improves outcomes consistently [36]; therefore, this is an area where active research is necessary in the future.

There are several potential weaknesses of our study alike other investigations involving RWD. The NHIFD includes detailed medical records of the whole population, but laboratory values concerning target LDL-C value achievement, the socioeconomic status of patients, and PCSK9i prescriptions were not available in the database. It is known from recent independent publications that LDL-C target achievement rate in post-ACS patients is about 11–20% in Central Europe, including Hungary. Taking into consideration that 32–50% of our patients discontinued LLT by the end of the observation period and that only 11% of those remaining on treatment received combined statin and ezetimibe therapy, this limitation does not influence the conclusions of our study. LDL-C goal attainment in the Hungarian population within the current administrative environment can only be assessed in a prospective study. Such a study would require enormous resources, and even if these were available, it is highly unlikely that the whole population could be covered. The lack of data on PCSK9i treatment was because these drugs were only reimbursed based on an individual approval by the NHIF after the submission of a request by the treating physician and the patient. Patients were eligible only for PCSK9i individual NHIF approval and prescription, if the target value was not reached on maximally tolerated statin + ezetimibe treatment. Therefore, the number of patients receiving PCSK9i therapy was negligible during the follow-up study period. Socioeconomic status could have influenced the treatment selection and persistence, but the potential effect of this parameter on the results is modest in our opinion. The cost of pharmaceuticals in general is 14–21% lower in Hungary than the EU average [37]. Measures implemented by the national authorities to increase generic competition, such as “blind bid” and generic prescription instead of branded prescription led to a significant reduction in the consumer price of statins [38]. In addition, national reimbursement regulations entitle post-ACS patients to 90% price compensation on the cost of ezetimibe. Thus, the price of combined high-intensity statin + ezetimibe can be as low as 1.6 EUR/month. A further aid is available for the most impoverished and socioeconomically deprived patients, the so-called “public healthcare”. Within this framework, patients with a valid public healthcare certificate can receive their lipid-lowering medicine free of charge from a predetermined monthly budget. Therefore, it can be stated that disadvantage alone does not prevent the secondary prevention patient population from accessing basic cardiovascular medicines in Hungary. Finally, it was shown in a study investigating the relationship between statin utilization and socioeconomic deprivation in Hungary that contrary to the common belief, statin prescription redemption was significantly higher in districts with the highest deprivation [39]. This unexpected but important finding was confirmed in a recent study from Sweden, examining socioeconomic disparities and mediators for recurrent ASCVD events after a first MI [40]. Socioeconomic status was assessed by disposable income quintile, educational level, and marital status. The researchers found that risk mediation through optimal statin management was negligible. Omitting cardiac rehabilitation, persistent smoking, poor blood pressure control, and cardiometabolic risk profile determined income-dependent prognosis after MI.

A strength of the HURDLE study was that it was a nation-wide survey of a significant number of ACS patients, who underwent revascularization in an Eastern European country in the year before the COVID-19 pandemic, with a follow-up time of at least 2 years. The insurance fund’s database allowed full-scale treatment follow-up and mortality analysis. No exclusion criteria were applied; thus, virtually every patient who was treated with PCI or CABG after an acute coronary event was enrolled. Selection bias did not affect the results of our study. Previous good-quality prospective trials generally involved highly selective patient populations from well-motivated centers, which might not reflect the general population. The COVID-19 pandemic impacted access to medical care and willingness to seek medical attention [41,42]; therefore, our results from before the COVID-19 era reflect a valid contemporary practice. It can be pointed out that the healthcare system and the historical, geographical, cultural, and social background of Central and Eastern European countries share several similarities. This is reflected by the epidemiology of ASCVD in this region. The total population of Eastern and Central European countries of the EU adds up to 94 million, which accounts for around 20% of the total EU population. Gaining information from this geographical area is, therefore, important for the healthcare development of not only Hungary but also for the European Union.

## 5. Conclusions

In the present study, several conclusions could be drawn up. These need to be addressed by policymakers and healthcare providers.

Firstly, the LLT is suboptimal. According to the Hungarian Myocardial Infarction Registry, statins were prescribed by the treating physician to 92% of patients who underwent PCI due to ACS in 2018. As shown in our follow-up data, 22% of these post-ACS patients were without this kind of treatment within 120 days after the index event. Moreover, every second patient discontinued LLT by 16 months after the index event. A smaller part of them should be declared statin intolerant (based on more than 4 million patients’ data, the rate of statin intolerance is 9.1% [43]), but the main real reason should be the patients’ adherence and physicians’ inertia [44].

Secondly, the results suggest that the lipid control of individuals with several comorbidities, females and elderly require closer attention. These groups seem to be vulnerable subpopulations with higher rates of multimorbidity, higher likelihood of undertreatment, higher mortality, and lower persistence.

Thirdly, this study also indicated that the effectiveness of lipid-lowering therapy might be improved by increasing the use of ezetimibe, where even with the current reimbursement regulations, we have significant resources to utilize. Additionally, novel lipid-lowering agents, such as PCSK9 inhibitors are likely to improve outcomes; therefore, these agents should be used more frequently. The reimbursement regulation for PCSK9 inhibitors in Hungary during our study made it difficult to use PCSK9 inhibitor treatment for most patients who did not reach the target value with maximum dose statin + ezetimibe treatment. To the benefit of our research, the new PCSK9i prescription rules implemented in 2024 have expanded this possibility. Hopefully, more and more patients who need it will soon be able to receive the effective PCSK9i treatment.

Multifactorial interventions are needed on behalf of healthcare providers, national policymakers, and patient support groups to enhance the more effective implementation of guidelines in real-life practice. This can reduce the number of recurring acute cardiovascular events and improve the patients’ life expectancies with better lipid control.

## Figures and Tables

**Table 1:** Definition of LLT intensity applied in the HURDLE study. LDL-C reduction is <30%, 30–50%, and >50% by the low-, moderate-, and high-intensity LLT regimens, respectively. N/A: non-applicable.

LLT	Low Intensity	Moderate Intensity	High Intensity
Atorvastatin	N/A	10–30 mg	40–80 mg
Ezetimibe	10 mg	N/A	N/A
Fluvastatin	20–40 mg	80 mg	N/A
Pravastatin	10–20 mg	40–80 mg	N/A
Rosuvastatin	N/A	5–15 mg	20–40 mg
Simvastatin	10 mg	20–40 mg	N/A

**Table 2:** Baseline characteristics, mean follow-up times, and comorbidities of Hungarian patients with incident ACS in 2018 on different lipid-lowering treatment regimens after the index event.

Baseline Characteristics and Comorbidities	Statin High Combination	Statin Moderate Combination	Statin Low Combination	Statin High	Statin Moderate	Ezetimibe Monotherapy
No.	312	77	193	9565	1624	188
Mean follow-up time (days, ±SD)	861 (±185.49)	852.8 (±194.77)	891.9 (±189,97)	870.6 (±210.3)	848.8 (±243.71)	891.9 (±189.97)
Age (y, mean ± SD)	63.9 (±10.75)	66.1 (±8.81)	67.0 (±10)	64.5 (±11.88)	68.6 (±11.68)	66.9 (±9.81)
Male, n (%)	192 (61.54)	46 (59.74)	112 (58.03)	6155 (64.35)	946 (58.25)	108 (57.45)
Heart failure, n (%)	68 (21.79)	22 (28.57)	54 (27.98)	1902 (19.88)	523 (32.2)	52 (27.66)
Peripheral arterial disease, n (%)	89 (28.53)	22 (28.57)	49 (25.39)	1758 (18.38)	431 (26.54)	48 (25.53)
Cerebrovascular disease, n (%)	88 (28.21)	30 (38.96)	55 (28.5)	1676 (17.52)	437 (26.91)	53 (28.19)
Diabetes, n (%)	136 (43.59)	43 (55.84)	84 (43.52)	2997 (31.33)	625 (38.49)	82 (43.62)
Chronic pulmonary disease, n (%)	74 (23.72)	16 (20.78)	44 (22.8)	1585 (16.57)	372 (22.91)	43 (22.87)
Hepatic disease, n (%)	20 (6.41)	7 (9.09)	12 (6.22)	304 (3.18)	92 (5.67)	12 (6.38)
Renal disease, n (%)	39 (12.5)	12 (15.58)	30 (15.54)	835 (8.73)	293 (18.04)	29 (15.43)
Gastrointestinal ulceration, n (%)	2 (0.64)	-	-	150 (1.57)	36 (2.22)	-
Tumor, n (%)	54 (17.31)	18 (23.38)	33 (17.1)	1548 (16.18)	344 (21.18)	32 (17.02)
Metastatic tumor, n (%)	2 (0.64)	-	-	82 (0.86)	13 (0.8)	-
Leukemia/lymphoma, n (%)	1 (0.32)	1 (1.3)	5 (2.59)	42 (0.44)	15 (0.92)	5 (2.66)
Lymphoma, n (%)	1 (0.32)	-	-	40 (0.42)	9 (0.55)	-
Connective tissue disease, n (%)	5 (1.6)	1 (1.3)	11 (5.7)	125 (1.31)	26 (1.6)	11 (5.85)
Alcohol use disorder, n (%)	-	2 (2.6)	1 (0.52)	203 (2.12)	47 (2.89)	1 (0.53)
Dementia/mental condition, n (%)	-	-	1 (0.52)	117 (1.22)	31 (1.91)	1 (0.53)
Mental condition, n (%)	45 (14.42)	15 (19.48)	27 (13.99)	1187 (12.41)	235 (14.47)	27 (14.36)

**Table 3:** Comparing the differences in baseline characteristics between patient groups on statin high combination (SHC), statin high (SH), and statin moderate (SM) therapy. Significant differences are highlighted.

Baseline Characteristics and Comorbidities	Statin High Combination (SHC)	Statin High (SH)	Statin Moderate (SM)	SHC vs. SH (*p*-Values)	SHC vs. SM (*p*-Values)	SH vs. SM (*p*-Values)
No.	312	9565	1624	-	-	-
Age (y, mean)	63.9	64.5	68.6	1.0000	0.0010	0.0010
Male (%)	61.54	64.35	58.25	1.0000	1.0000	<0.0001
Heart failure (%)	21.79	19.88	32.2	1.0000	0.0026	<0.0001
Peripheral arterial disease (%)	28.53	18.38	26.54	<0.0001	1.0000	<0.0001
Cerebrovascular disease (%)	28.21	17.52	26.91	<0.0001	1.0000	<0.0001
Diabetes (%)	43.59	31.33	38.49	<0.0001	0.9089	<0.0001
Chronic pulmonary disease (%)	23.72	16.57	22.91	0.0089	1.0000	<0.0001
Hepatic disease (%)	6.41	3.18	5.67	0.0161	1.0000	<0.0001
Renal disease (%)	12.5	8.73	18.04	0.2102	0.1738	<0.0001
Gastrointestinal ulceration (%)	0.64	1.57	2.22	1.0000	0.5950	0.5289
Tumor (%)	17.31	16.18	21.18	1.0000	1.0000	<0.0001
Metastatic tumor (%)	0.64	0.86	0.8	1.0000	1.0000	1.0000
Leukemia/lymphoma (%)	0.32	0.44	0.92	1.0000	1.0000	0.1121
Lymphoma (%)	0.32	0.42	0.55	1.0000	1.0000	1.0000
Connective tissue disease (%)	1.6	1.31	1.6	1.0000	1.0000	1.0000
Alcohol use disorder (%)	-	2.12	2.89	0.0932	0.0235	0.5170
Dementia/mental condition (%)	-	1.22	1.91	0.4445	0.1250	0.2281
Mental condition (%)	14.42	12.41	14.47	1.0000	1.0000	0.2116

**Table 4:** One-year adherence to different lipid-lowering therapies, as well as their one- and two-year persistence (assuming a 60-day grace period).

Lipid-Lowering Therapy	Adherence	Persistence 1-Year	Persistence 2-Year
Moderate-dose statin	56%	52%	38%
Ezetimibe mono and low-dose statin	61%	57%	48%
High-dose statin	63%	59%	45%
Combination, moderate-dose statin	66%	66%	45%
Combination, high-dose statin	69%	67%	54%

## Data Availability

The raw data supporting the conclusions of this article will be made available by the authors upon request.

## Supplementary Materials

The following supporting information can be downloaded at: https://www.mdpi.com/article/10.3390/jcm13216562/s1, Table S1: Baseline characteristics and comorbidities of Hungarian patients with incident ACS in 2018 receiving lipid-lowering treatment after the index event. Figure S1: Persistence of lipid-lowering therapy in post-ACS male patients across different therapeutic regimens by 60-day grace period. Selected time points are presented in numerical format (with 95% confidence intervals) for illustration. Figure S2: Persistence of lipid-lowering therapy in post-ACS female patients across different therapeutic regimens by 60-day grace period. Selected time points are presented in numerical format (with 95% confidence intervals) for illustration. Figure S3: Persistence of lipid-lowering therapy in post-ACS 0–59-year-old patients across different therapeutic regimens by 60-day grace period. Selected time points are presented in numerical format (with 95% confidence intervals) for illustration. Figure S4: Persistence of lipid-lowering therapy in post-ACS >=60-year-old patients across different therapeutic regimens in a 60-day grace period. Selected time points are presented in numerical format (with 95% confidence intervals) for illustration. Figure S5: Survival analysis of male patients on different lipid-lowering therapeutic regimens during the follow-up period. Selected time points are presented in numerical format (with 95% confidence intervals) for illustration. Figure S6: Survival analysis of female patients on different lipid-lowering therapeutic regimens during the follow-up period. Selected time points are presented in numerical format (with 95% confidence intervals) for illustration. Figure S7: Survival analysis of 0–59-year-old patients on different lipid-lowering therapeutic regimens during the follow-up period. Selected time points are presented in numerical format (with 95% confidence intervals) for illustration. Figure S8: Survival analysis of >=60-year-old patients on different lipid-lowering therapeutic regimens during the follow-up period. Selected time points are presented in numerical format (with 95% confidence intervals) for illustration. Figure S9: Persistence (a,b,c) and survival analysis (d,e,f) curves of patients on different lipid-lowering therapeutic regimens, where a significant difference was found between the compared prespecified treatment groups. The dotted lines illustrate 95% confidence intervals (CIs). Selected time points are presented in numerical format (with 95% CI) for illustration. Green arrows represent relative risk reduction (RRR) for therapeutic nonadherence (a,b,c) or mortality (d,e,f) between the compared therapeutic regimens at 12 and 24 months.
