# Supplementary material for: A Nation-Wide Evaluation of Suboptimal Lipid-Lowering Treatment Patterns Among Patients Undergoing Intervention for Acute Coronary Syndrome in Hungary
Source: J Clin Med. 2024 Oct 31;13(21):6562. doi: 10.3390/jcm13216562 (PMC11547159; doi:10.3390/jcm13216562)
Supplement: Supplementary file 1 [file jcm-13-06562-s001.zip › Table S1.pdf]

**Baseline Characteristics and Comorbidities (n = 11,959)**	**Number**	**Percent (%)**
Mean follow-up time (days, mean)	868	
Age (years, mean)	65.1	
Male (n)	7559	63,2
Heart failure (n)	2621	21,9
Peripheral arterial disease (n)	2397	20,0
Cerebrovascular disease (n)	2339	19,6
Diabetes (n)	3967	33,2
Chronic pulmonary disease (n)	2134	17,8
Hepatic disease (n)	447	3,7
Renal disease (n)	1238	10,4
Gastrointestinal ulceration (n)	188	1,6
Tumor (n)	2029	17,0
Metastatic tumor (n)	97	0,8
Leukemia/lymphoma (n)	69	0,6
Lymphoma (n)	50	0,4
Connective tissue disease (n)	179	1,5
Alcohol use disorder (n)	254	2,1
Dementia/mental condition (n)	150	1,3
Mental condition (n)	1536	12,8
