# Supplementary figures and images for: A Nation-Wide Evaluation of Suboptimal Lipid-Lowering Treatment Patterns Among Patients Undergoing Intervention for Acute Coronary Syndrome in Hungary
Source: J Clin Med. 2024 Oct 31;13(21):6562. doi: 10.3390/jcm13216562 (PMC11547159; doi:10.3390/jcm13216562)

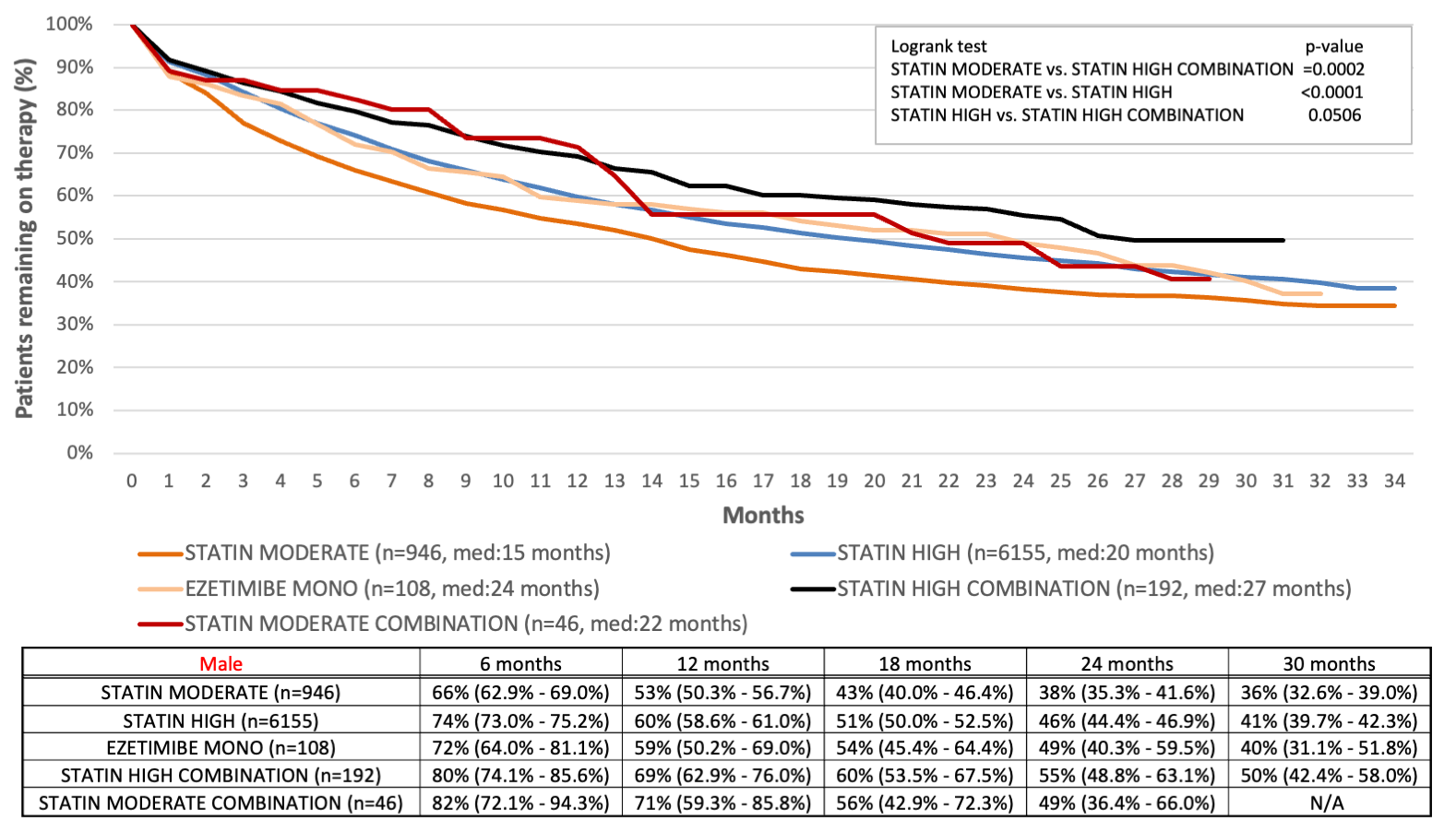

Supplement: Supplementary file 1 [file jcm-13-06562-s001.zip › Supplementary Figure 1.tiff]

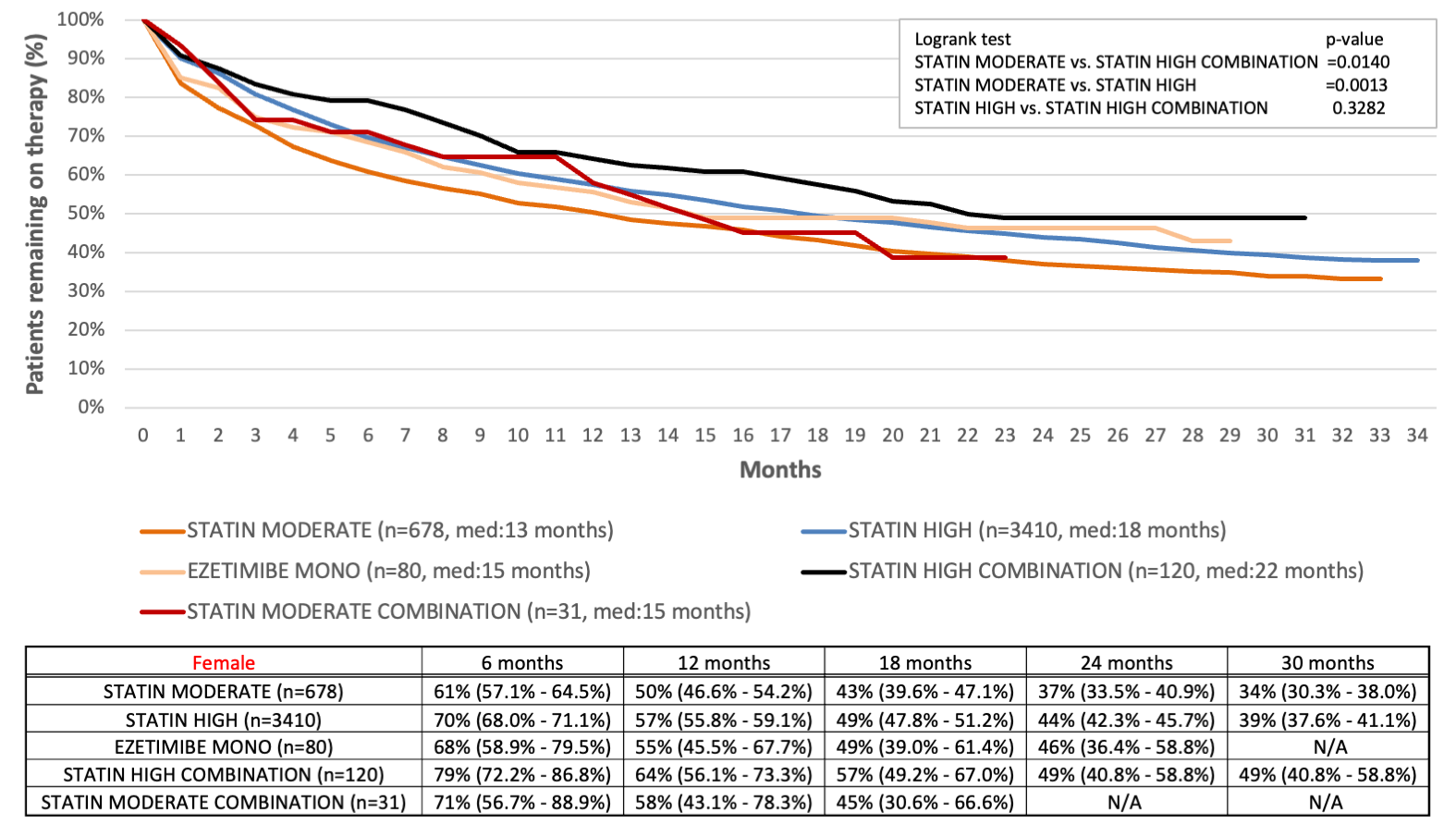

Supplement: Supplementary file 1 [file jcm-13-06562-s001.zip › Supplementary Figure 2.tiff]

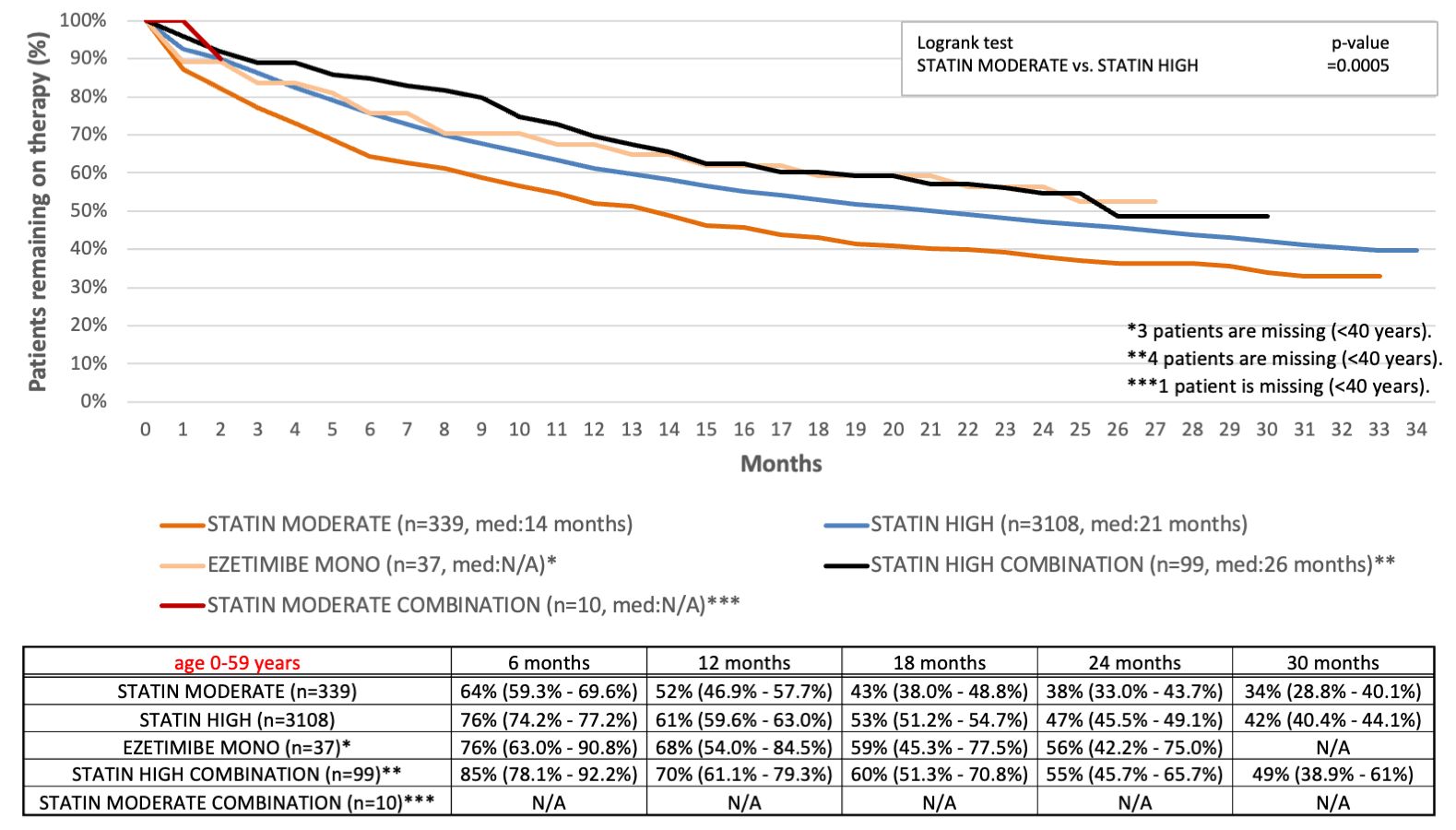

Supplement: Supplementary file 1 [file jcm-13-06562-s001.zip › Supplementary Figure 3.tiff]

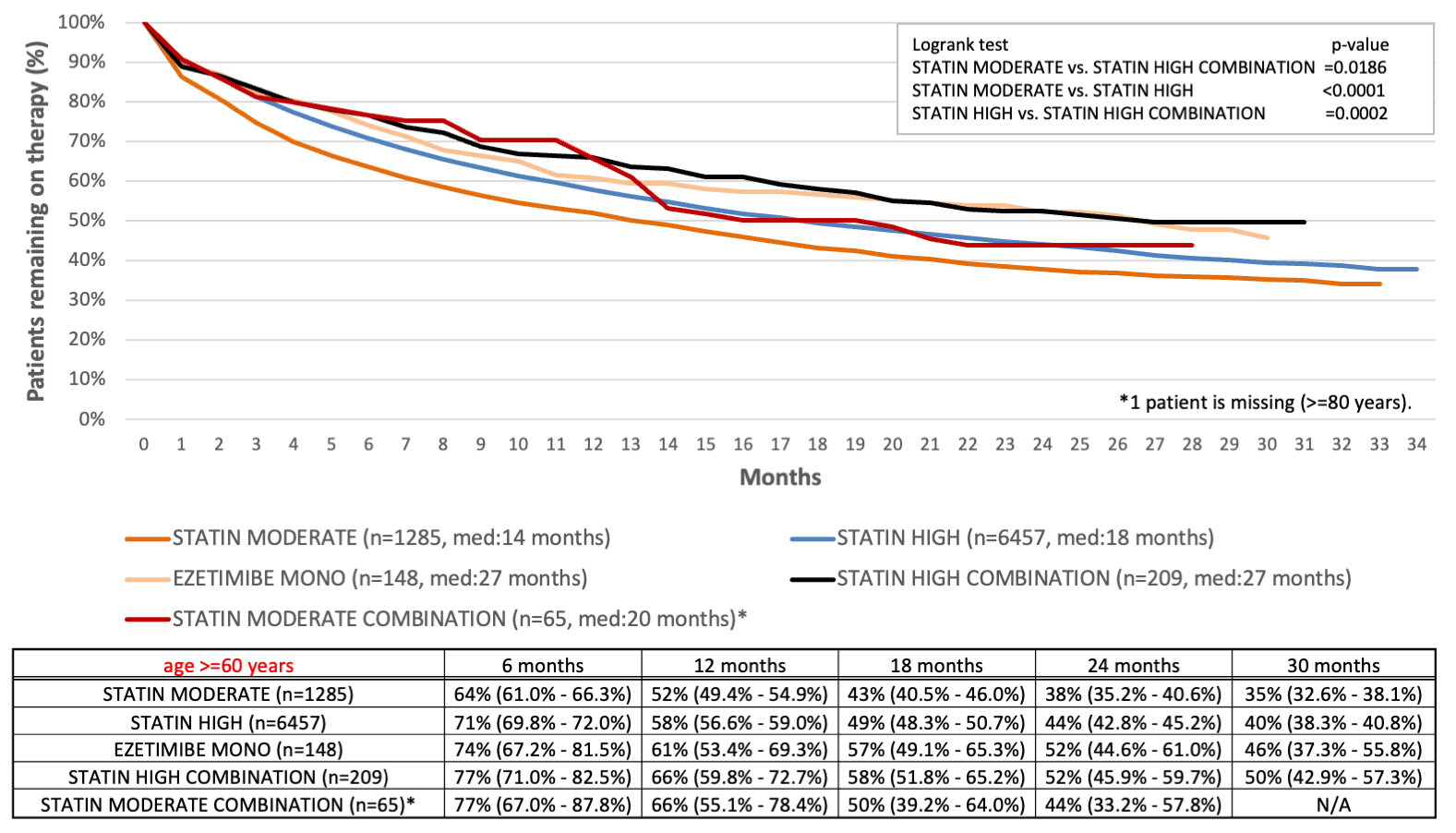

Supplement: Supplementary file 1 [file jcm-13-06562-s001.zip › Supplementary Figure 4.tiff]

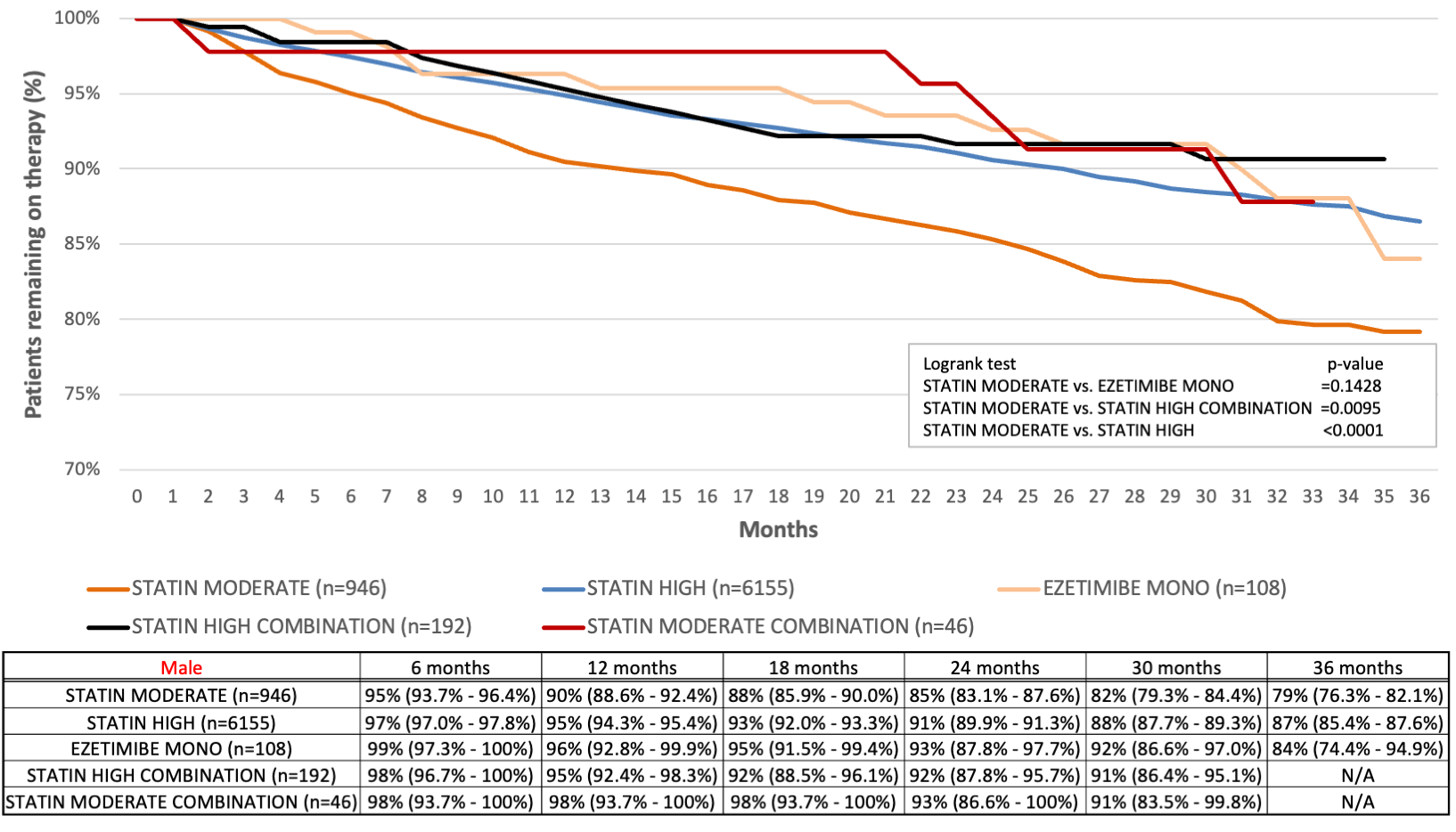

Supplement: Supplementary file 1 [file jcm-13-06562-s001.zip › Supplementary Figure 5.tiff]

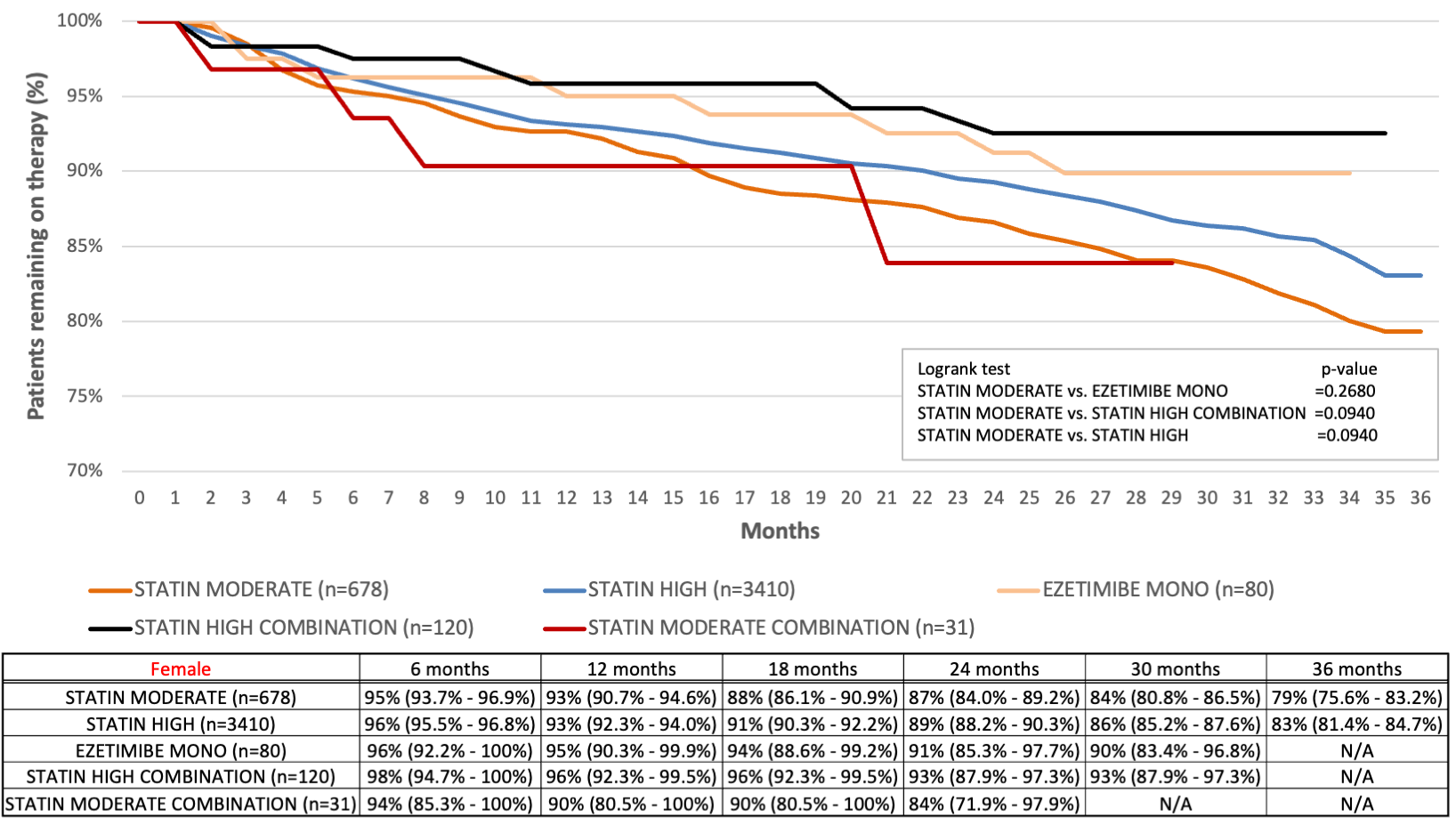

Supplement: Supplementary file 1 [file jcm-13-06562-s001.zip › Supplementary Figure 6.tiff]

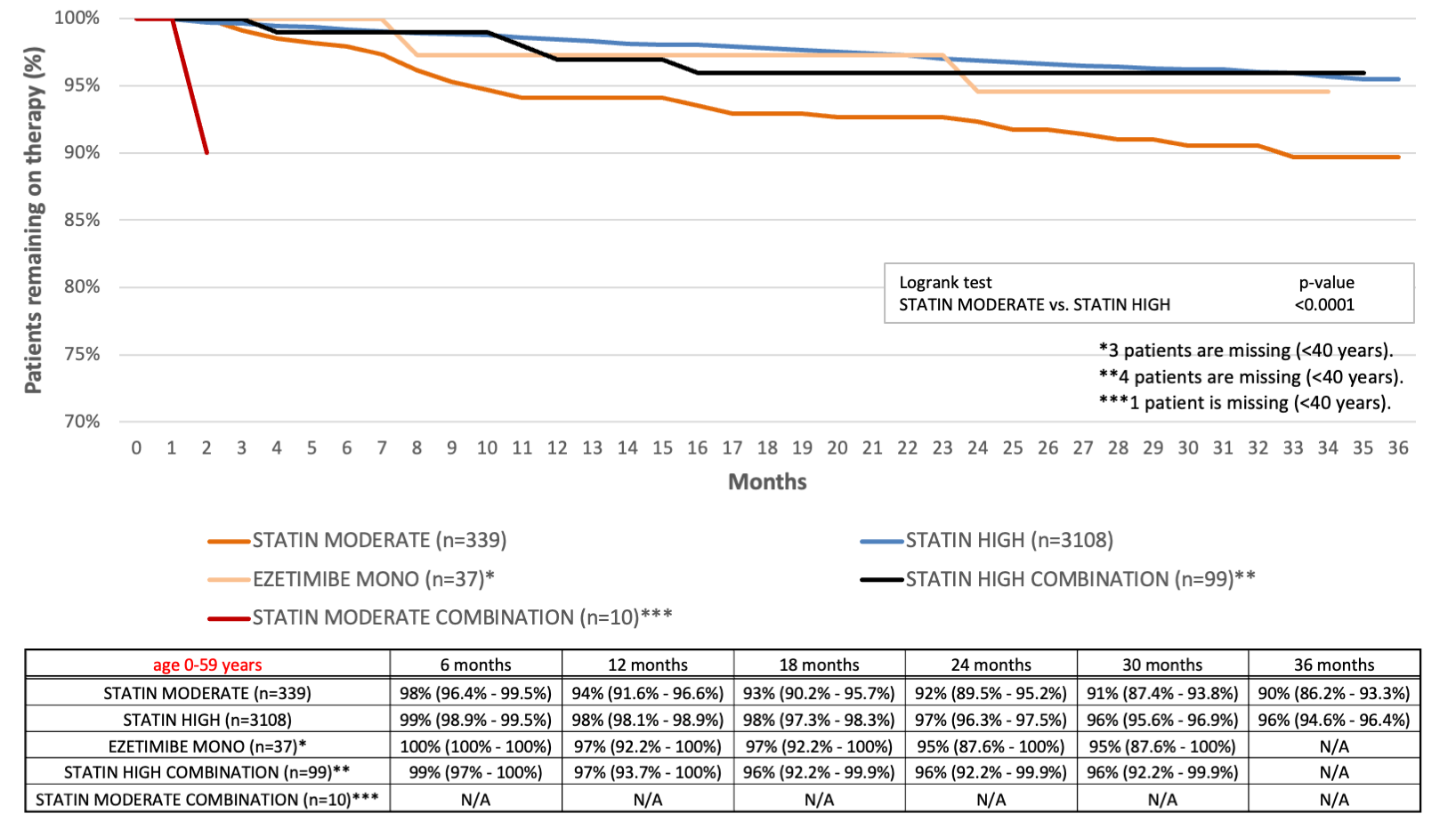

Supplement: Supplementary file 1 [file jcm-13-06562-s001.zip › Supplementary Figure 7.tiff]

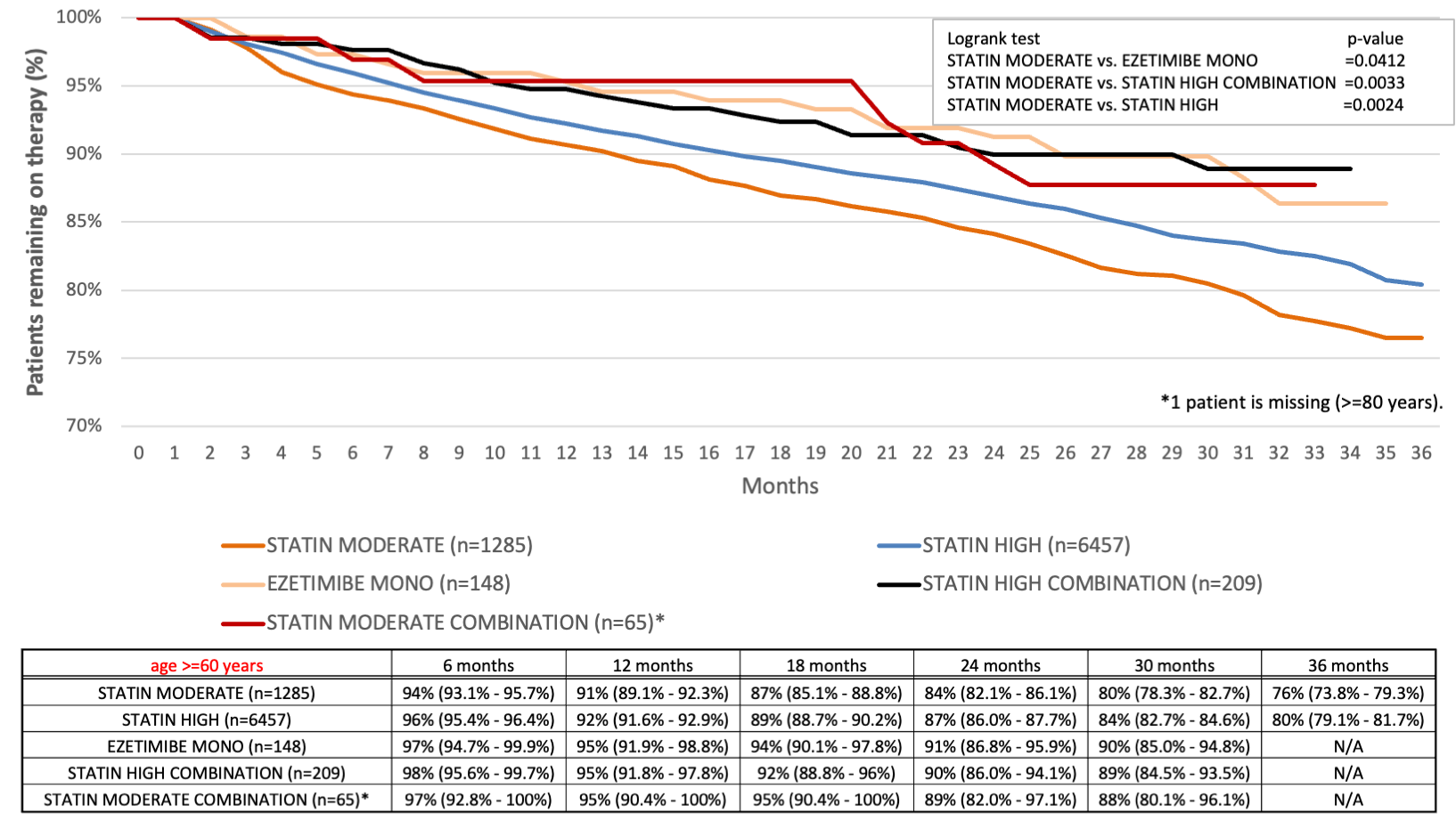

Supplement: Supplementary file 1 [file jcm-13-06562-s001.zip › Supplementary Figure 8.tiff]

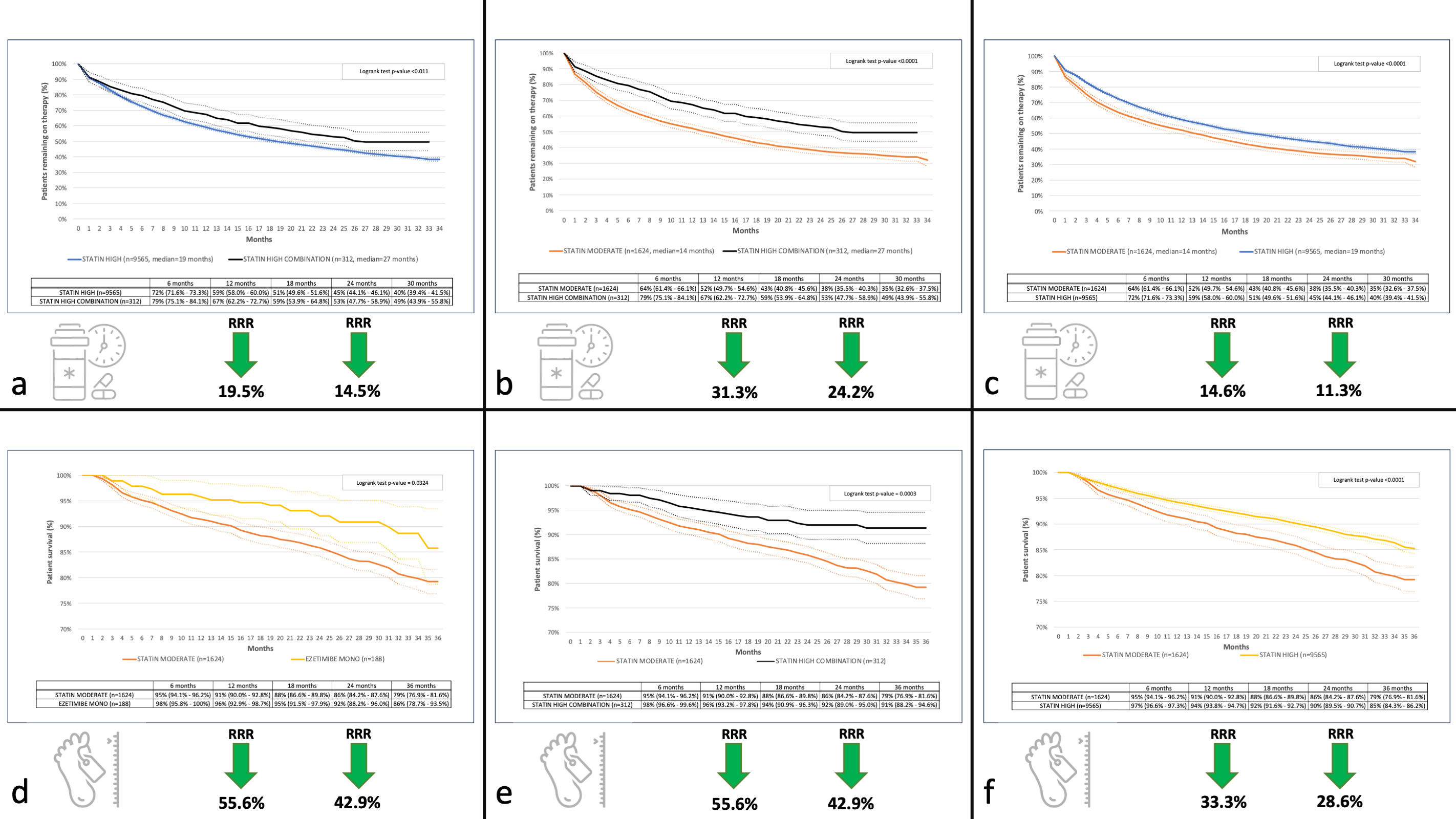

Supplement: Supplementary file 1 [file jcm-13-06562-s001.zip › Supplementary Figure 9.tiff]
